# Design of Acetaldehyde Gas Sensor Based on Piezoelectric Multilayer Microelectromechanical System Resonator

**DOI:** 10.3390/mi15080962

**Published:** 2024-07-28

**Authors:** Primavera Argüelles-Lucho, Rosa M. Woo-García, Leandro García-González, Rene Pérez-Cuapio, Natiely Hernández-Sebastian, Agustín L. Herrera-May, Francisco López-Huerta

**Affiliations:** 1 Facultad de Ingeniería de la Construcción y el Hábitat, Universidad Veracruzana, Boca del Río 94294, Veracruz, Mexico; prarlu@gmail.com (P.A.-L.); rwoo@uv.mx (R.M.W.-G.); 2 Tecnológico Nacional de México, Campus Veracruz, Veracruz 91800, Veracruz, Mexico; 3 Faculty of Electrical and Electronic Engineering, University Veracruzana, Boca del Río 94294, Veracruz, Mexico; 4 Micro and Nanotechnology Research Center, Universidad Veracruzana, Boca del Río 94294, Veracruz, Mexico; leagarcia@uv.mx; 5 Faculty of Chemical Engineering, Benemerite Autonomous University of Puebla, Puebla 72570, Puebla, Mexico; reneperezcuapio@gmail.com; 6 Optical Research Center, León 37150, Guanajuato, Mexico; natiely@cio.mx

**Keywords:** acetaldehyde, gas sensor, piezoelectric, MEMS, resonator, multilayer

## Abstract

Acetaldehyde is a volatile organic compound that can cause damage at the cellular and genomic levels. The monitoring of acetaldehyde gas at low concentrations requires fast-response and low-cost sensors. Herein, we propose the design of an acetaldehyde gas sensor based on a low-cost Microelectromechanical System (MEMS) process. This sensor is formed by a single-clamped piezoelectric multilayer resonator (3000 × 1000 × 52.2 µm) with a simple operating principle and easy signal processing. This resonator uses a zinc oxide piezoelectric layer (1 µm thick) and a sensing film of titanium oxide (1 µm thick). In addition, the resonator uses a support layer of 304 stainless steel (50 µm thick) and two aluminum layers (100 nm thick). Analytical and Finite-Element Method (FEM) models are developed to predict the mechanical behavior of the gas sensor, considering the influence of the different layers of the resonator. The analytical results agree well with respect to the FEM model results. The gas sensor has a first bending frequency of 4722.4 Hz and a sensitivity of 8.22 kHz/g. A minimum detectable concentration of acetaldehyde of 102 ppm can be detected with the proposed sensor. This gas sensor has a linear behavior to detect different acetaldehyde concentrations using the frequency shifts of its multilayer resonator. The gas sensor design offers advantages such as small size, a light weight, and cost-efficient fabrication.

## 1. Introduction

Air pollution from volatile organic compound (VOC) emissions can cause respiratory diseases and deaths [1]. Acetaldehyde is a volatile organic compound used to fabricate acetic acid, flavorings, aniline dyes, plastics, and synthetic rubber [2]. Toxic acetaldehyde gas can generate drowsiness, fainting, and cellular damage [3,4]. Thus, fast-response sensors are required to detect low concentrations of acetaldehyde gas in the environment. Microelectromechanical System (MEMS) technology is an alternative to developing these sensors. This technology can allow gas sensors with important advantages such as small size, fast response, portability, light weight, mass-fabrication process, low power consumption, and high sensibility [5,6,7]. MEMS-based gas sensors can use resonators to monitor the gas concentration as a function of the variation of their resonant frequencies [8,9,10]. This frequency shift can be detected using transduction mechanisms such as piezoresistive, optical, capacitive, and piezoelectric sensing [11,12,13,14]. The piezoresistive sensors are suitable for bulk micromachining processes and have easy signal processing. However, this sensing technique has high temperature dependence and requires temperature compensation components. On the other hand, the optical method permits gas sensors with minimum electronic elements, which are not affected by electromagnetic interference. Furthermore, these sensors incorporate optical systems that can increase their size and cost.

Generally, capacitive gas sensors are fabricated using superficial micromachining processes that can integrate electronic components on the same chip. Thus, capacitive gas sensors have advantages such as small size, light weight, and minimum influence on temperature variations. Nevertheless, the performance of these sensors is affected by air damping. This air damping is decreased when the capacitive gas sensors are vacuum-packaged. Another sensing technique is piezoelectric sensing, which has advantages such as a low-cost fabrication process, no complex signal processing, simple structural configurations, and low power consumption. These advantages can allow the development of novel MEMS-based gas sensors to monitor acetaldehyde in different environments.

Nakate et al. [15] fabricated an acetaldehyde gas sensor based on p-n heterojunction interface of NiO nanosheets into WO_3_ nanorods. This sensor exhibited a high response and selectivity towards acetaldehyde gas. In addition, this sensor had a high response to acetaldehyde concentrations between 20 and 100 ppm. Thomas et al. [16] developed Co-doped (1−5 wt%) ZnO films deposited on glass substrates using spray pyrolysis. These films had a very high response toward acetaldehyde concentrations. The 5 wt% Co-doped ZnO film can detect 5 ppm of acetaldehyde at 300 K due to Co’s catalytic properties. This film has potential applications in acetaldehyde gas sensors. Jin et al. [17] reported a n−n heterojunction sensor composed of poly(3,4-ethylenedioxythiophene): polystyrenesulfonate (PEDOT:PSS) doped with MoS_2_ quantum dots (QDs). The PEDOT:PSS composite doped with 20 wt% MoS_2_ QDs achieved a detection limit of 1 ppm of acetaldehyde concentration. However, the sensitivity and resolution of these gas sensors can be significantly affected by the weight percent of the doped materials. Furthermore, these gas sensors require a complex and well-controlled fabrication process. 

Hajjam and Pourkamali [18] proposed a MEMS-based resonant organic gas sensor composed of a thermal–piezoresistive resonator. This resonator was fabricated using a standard silicon-on-insulator (SOI) MEMS fabrication process. A polymer coating on the surface of the resonator was required to characterize the performance of this resonator as a VOC sensor. This sensor measured a minimum detectable concentration of toluene close to 4.8 ppm. However, this sensor requires a complex fabrication process and a polymer coating on the resonator surface, which can cause excessive polymer residues and damage the performance of the resonator. Li et al. [19] designed a virtual sensor array using an AlN piezoelectric resonator. This resonator included five groups of top electrodes to detect several VOCs based on frequency variations of different vibration modes and changes in impedance magnitudes. This resonator was fabricated using an SOI wafer that required an AlN film deposited on the resonator and a sensing layer of graphene oxide. This sensor registered a limit of detection to ethanol concentration of 25 ppm. Arabi et al. [20] implemented MEMS sensors to identify hydrogen sulfide and formaldehyde VOCs. This sensor has a resonator formed by two cantilevers and a plate fabricated using the surface micromachining process PolyMUMPs. Two different polymeric materials (emeraldine polyaniline hydrochloride and poly(2,5−dimethyl aniline)) were employed as sensing layers to VOCs. A minimum detectable concentration of formaldehyde of 1 ppm can be measured using this sensor. Nevertheless, these MEMS-based gas sensors were developed using expensive and complex fabrication processes. In order to overcome this problem, we designed a novel acetaldehyde gas sensor based on a low-cost MEMS fabrication process. This sensor is composed of a piezoelectric multilayer resonator (3000 µm × 1000 µm × 52.2 µm) with a simple operating principle and easy signal processing, which can be developed without requiring high temperatures in comparison with conventional micromachining processes based on silicon. This sensor uses a sensing layer of TiO_2_ film to detect acetaldehyde concentrations. In addition, this resonator employs a support layer of 304 stainless steel (50 µm thickness), two aluminum layers (100 nm thickness), a ZnO layer (1 µm thickness), and a TiO_2_ film (1 µm thickness). We use the support layer based on 304 stainless steel due to it having high mechanical properties and its ability to be etched with a non-expensive fabrication process. Our gas sensor has a linear behavior for detecting different acetaldehyde concentrations using the frequency shifts of its resonator. Also, we proposed analytical models based on the Macaulay and Rayleigh methods to study the deflection and first bending resonant frequency of the piezoelectric multilayer resonator. These models included the effect of the different layers of the resonator on its mechanical behavior. Finite-Element Method (FEM) models of the resonator were developed to predict its dynamic structural response. The design of our gas sensor can detect a minimum acetaldehyde concentration of 102 ppm. This proposed gas sensor has advantages such as small size, light weight, and cost-effective fabrication.

This work is organized as follows: Section 2 describes the analytical models used to determine the displacement and first bending resonant frequency of the resonator of the acetaldehyde gas sensor. In addition, this section considers the FEM models of the resonator to predict the electromechanical behavior of the resonator. Section 3 reports the results of the performance of the gas sensor based on the analytical and FEM models. Finally, Section 4 includes the conclusions and future research work.

## 2. Materials and Methods

This section describes the analytical models to determine the displacement and first bending resonant frequency of the resonator of the acetaldehyde gas sensor. In addition, this section considers the FEM models of the resonator to predict the electromechanical behavior of the resonator. 

### 2.1. Design and Modeling

**Figure 1:**
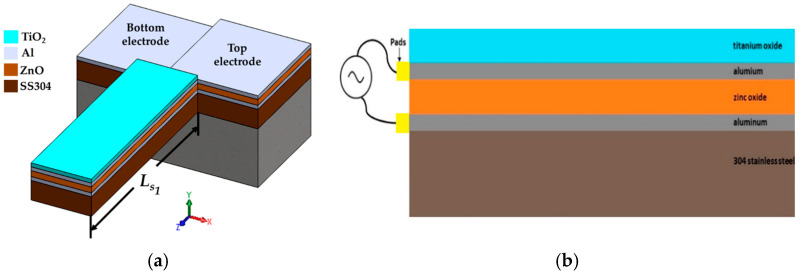
Design of the acetaldehyde gas sensor. (**a**) Three-dimensional view of the main components and materials of the sensor and (**b**) a cross-section view of the different layers of the gas sensor.

**Figure 2:**
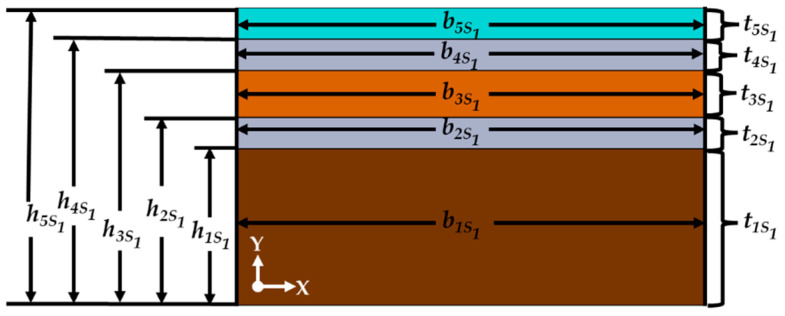
Geometrical parameters of the different layers of the acetaldehyde gas sensor.

Figure 1 depicts the different structural components of the design of the acetaldehyde gas sensor. Figure 2 shows the main geometrical parameters of the different layers of the sensor design. These parameters are used for modeling the mechanical performance of the sensor. We use the Rayleigh and Macaulay methods, as well as the Euler–Bernoulli beam theory.

We employ the Rayleigh method [21,22] to predict the first bending resonant frequency of the structure of the gas sensor. This method is based on the principle of conservation of energy, which determines that the maximum kinetic energy *K_m_* and the maximum potential energy *P_m_* of a structure have the same value. For a single-clamped beam, the *P_m_* and *K_m_* can be obtained as:

(1) $$P_{m}=\frac{1}{2}\int_{0}^{L}EI(x){\left(\frac{\partial^{2}y(x)}{\partial x^{2}}\right)}^{2}dx$$

(2) $$K_{m}=\frac{{\left(2\pi f\right)}^{2}}{2}\int_{0}^{L}\rho A(x)y^{2}(x)dx$$

where *y*(*x*) is the deflection and *I* is the moment of inertia of the cross-section along the beam of length *L*, *ρ* is the density, *E* is the Young’s modulus of the beam material, *A* is the area of the cross-section of the beam calculated as *bt*, *b* is the width of the beam, and *t* is the thickness of the beam. *EI* is the bending stiffness of the resonator that depends on *b* and *t*. By equating *P_m_* = *K_m_*, the first bending resonant frequency (*f_r_*) of the acetaldehyde gas sensor can be determined as follows:

(3) $$f_{r}=\frac{1}{2\pi }\sqrt{\frac{\int_{0}^{L}EI\left(x\right){\left(\frac{\partial^{2}y\left(x\right)}{\partial x^{2}}\right)}^{2}dx}{\int_{0}^{L}\rho A\left(x\right)y^{2}\left(x\right)dx}}$$

We used the Rayleigh–Ritz method to obtain the first bending resonant frequency of the gas sensor. Therefore, the following assumptions were considered for the mathematical model: the materials are homogeneous and isotropic, the geometry of the gas sensor is symmetric with the *xy* plane, and the residual stress, nonlinearity, and surface effects of the resonant structure were neglected. The Euler–Bernoulli beam theory considers that the resonator must have an aspect ratio (length/thickness) greater than 10.

Table 1 indicates the properties of the materials, which are used in the mathematical model of the gas sensor to calculate its elastic centroid *a_sj_*, bending rigidity *EI_z_*, potential energy *P_m_*, kinetic energy *K_m_*, resonant frequency *f_r_*, quality factor *Q,* and damping ratio ζ [23,24]:

(4) $$a_{Sj}=\frac{{\left(ES\right)}_{Sj}}{{\left(EA\right)}_{Sj}}=\frac{\iint_{A_{Sj}}E_{Sj}y_{Sj}(x)dydz}{\iint_{A_{Sj}}E_{Sj}dydz}=\frac{1}{2}\frac{\sum_{i=1}^{q}E_{iSj}b_{iSj}t_{iSj}\left(h_{iSj}+h_{(i-1)Sj}\right)}{\sum_{i=1}^{q}E_{iSj}b_{iSj}t_{iSj}}$$

where *A_Sj_* is the domain in the *j*th section, *E_iSj_* is the Young’s modulus of the *i*th film placed in the *j*th section, *b_iSj_* is the width of the *i*th film located in the *j*th section, *t_iSj_* is the thickness of the *i*th film located in the *j*th section, and *h*_(*i*−1)*Sj*_ is the distance between the lower surface of the first film and the upper surface of the film (*i* − 1) located in the *j*th section. The bending rigidity (*EI_z_*) of the gas sensor can be determined with Equation (5):

(5) $${\left(EI_{z}\right)}_{S_{j}}=\sum_{i}^{q}{\left(E_{i}I_{zi}\right)}_{S_{j}}=\iint_{A_{S_{j}}}E_{S_{j}}y_{S_{j}}\left(x\right)dy=\frac{1}{3}\sum_{i=1}^{q}E_{iS_{j}}b_{iS_{j}}\left[{\left(h_{iS_{j}}-a_{S_{j}}\right)}^{3}-{\left(h_{(i-1)S_{j}}-a_{S_{j}}\right)}^{3}\right]$$

The methods used to determine the deflection of the gas sensor are the Euler–Bernoulli beam theory and the Macaulay method. This method describes the load types on structures with variable cross-sections [25]. The static deflection *y_S_*_1_ of the resonator of the acetaldehyde gas sensor, considering one section, is determined by:

(6) $${\left({EI}_{z}\right)}_{S_{1}}\frac{\partial^{2}y_{S_{1}}(x)}{\partial x^{2}}=M_{S_{1}}(x)\;\;0<x<L_{S_{1}}$$

$L_{S_{1}}$ is the length of the first section of the resonator of the gas sensor, as shown in Figure 1a.

The boundary condition of the resonator of the gas sensor is given by:

(7) $$y_{S1}(0)=0\;\;\frac{\partial y_{S1}(0)}{\partial x}=0$$

The bending moment of the *j*th section *M_S__j_* of the resonator is obtained by integrating the load function of the resonator twice, determined by the Macaulay method. The total load function *q*(*x*) is calculated as:

(8) $$q(x)=-M_{0}{\langle x-0\rangle }^{-2}+R_{0}{\langle x-0\rangle }^{-1}-\omega_{S1}{\langle x-0\rangle }^{0}+\omega_{S1}{\langle x-L_{S1}\rangle }^{0}$$

where *R*_0_, *M*_0_, and *ω_Sj_* are determined as:

(9) $$\omega_{Sj}=\rho_{iSj}gb_{iSj}t_{iSj}$$

(10) $$M_{0}=\omega_{Sj}L_{Sj}\left(\frac{1}{2}L_{Sj}\right)$$

(11) $$R_{0}=\omega_{Sj}L_{Sj}$$

The weight per unit length of the *j*th section is represented by *ω_Sj_* and the gravitational acceleration is represented by *g*. The *b_iSj_* takes into account the total width of all layers located at *h_iSj_* of the first section. By integrating Equation (8) with respect to *x* and considering the integration rules of Macaulay’s functions, the shear load function *V*(*x*) is expressed as:

(12) $$V(x)=-M_{0}{\langle x-0\rangle }^{-1}+R_{0}{\langle x-0\rangle }^{0}-\omega_{S1}{\langle x-0\rangle }^{1}+\omega_{S1}{\langle x-L_{S1}\rangle }^{1}+C_{1}$$

By integrating Equation (12) with respect to *x*, the function of the bending moment *M*_(*x*)_ of the resonator is calculated as:

(13) $$M(x)=-M_{0}{\langle x-0\rangle }^{0}+R_{0}{\langle x-0\rangle }^{1}-\frac{1}{2}\omega_{S1}{\langle x-0\rangle }^{2}+\frac{1}{2}\omega_{S1}{\langle x-L_{S1}\rangle }^{1}+C_{1}+C_{2}$$

The integration constants *C*_1_ and *C*_2_ are determined by the contour conditions *V*(0) = *R*_0_ y *M*(0) = *M*_0_ when replacing these conditions in Equations (12) and (13), obtaining *C*_1_ = *C*_2_ = 0. Thus, the bending moment of the first multilayer section of the resonator of the acetaldehyde gas sensor is obtained.

For $0<x<L_{S_{1}}$

(14) $$M_{S_{1}}(x)=-M_{0}{\langle x-0\rangle }^{0}+R_{0}{\langle x-0\rangle }^{1}-\frac{1}{2}\omega_{S_{1}}{\langle x-0\rangle }^{2}$$

In order to determine the deflection of the resonator of the gas sensor, Equation (14) is substituted into Equation (6), and double integration is used, applying the integration rules of the Macaulay function [30]. In addition, the boundary conditions of Equation (7) are regarded. Thus, the static deflection *y_S_*_1_(*x*) of the first section of the resonator of the gas sensor is calculated as:

For $0<x<L_{S1}$

(15) $$y_{S1}(x)=\frac{1}{{\left(EI_{z}\right)}_{S1}}\left[-\frac{1}{2}M_{0}{\langle x-0\rangle }^{2}+\frac{1}{6}R_{0}{\langle x-0\rangle }^{3}-\frac{1}{24}\omega_{S1}{\langle x-0\rangle }^{4}\right]$$

The first resonant frequency of the acetaldehyde gas sensor is obtained by substituting Equation (15) into Equations (1)–(3). Our analytical model to determine this resonant frequency of the sensor included the effect of the different layers of the resonator. Thus, the relative error of the first bending resonant frequency of the resonator can be reduced. In addition, Equation (15) can be employed to estimate the influence of all the layers of the resonator on the behavior of its static deflection. It can be suitable for the optimization process of the sensor design.

On the other hand, the first bending resonant frequency (*f_r_*) of the gas sensor changes due to the acetaldehyde mass (Δ*m_c_*) deposited on the upper surface of the resonator:

(16) $$f_{r}=\frac{1}{2\pi }\sqrt{\frac{k}{m_{e}+\Delta m_{c}}}$$

where *K* is the stiffness of the resonator and *m_e_* is the equivalent mass of the resonator.

The sensibility (*S*) of the gas sensor can be determined as the variation of its first bending resonant frequency (Δ*f_r_*) due to the changes in the acetaldehyde mass (Δ*m_c_*) [31]:

(17) $$S=\frac{\Delta f_{r}}{\Delta m_{c}}$$

By using Equations (16) and (17), we obtained the following equation:

(18) $$\frac{\Delta f_{r}}{f_{r}}=\frac{1}{2}\frac{\Delta m_{c}}{m_{e}}$$

The mass change in acetaldehyde can be estimated by:

(19) $$\Delta m_{c}=2m_{e}\frac{\Delta f_{r}}{f_{r}}$$

On the other hand, the following equation predicts the parts per million (ppm) of acetaldehyde using the gas sensor [32]:

(20) $$ppm=\frac{VM}{PM}C$$

where *VM* is the molar volume of the acetaldehyde, *PM* is the molecular weight of acetaldehyde, and *C* is the acetaldehyde concentration in mg/m^3^.

According to Equation (19), the equivalent mass of the resonator is 29.03 µg, and for a frequency step of 20 Hz and a first mode frequency of 4774.10 Hz, a mass change of 2432.32 ng is obtained; to calculate the parts per million, the concentration of acetaldehyde in the gas chamber must be calculated according to the following expression:

$$C=\frac{m_{e}}{V}$$

where V is the volume of the gas chamber in which the measurements were made (a tubular gas chamber with an internal diameter of 64 cm and a length of 40 cm, made of polytetrafluoroethylene material, was designed and constructed), giving a volume of 0.13 m^3^. The molar volume (MV) can be calculated by:

(21) $$VM=\frac{nRT}{P}$$

where n is the number of moles, R is the universal gas constant, T is the absolute temperature, and P is the pressure. The values used for Equation (20) are a PM of 44.05 g/mol, VM of 240.84 L/mol, and acetaldehyde concentration of 18.70 × 10^−3^ mg/m^3^, which results in 102 ppm. With these values, it is possible to detect frequency variations up to 5 Hz with a high-resolution digital frequency meter [33].

Air damping is the main damping source of the gas sensor, which is related to the quality factor (*Q*) of the resonant structure of the sensor. This quality factor is the relationship between the total energy stored in the gas sensor (*E_T_*) and the factor of energy lost per cycle (*E_c_*) caused by the damping source. This factor is affected by the air pressure around the gas sensor [26].

(22) $$Q=2\pi \frac{E_{T}}{E_{C}}$$

The damping ratio (ζ) of the resonator depends on its quality factor and it can be calculated by:

(23) $$\zeta =\frac{1}{2Q}$$

Table 2 contains the geometric parameters of the gas sensor, which are used in its analytical modeling. The bending stiffness (*EI_z_*), reaction load (*R*_0_), and bending moment (*M*_0_) of the resonator of the gas sensor are shown in Table 3. By using Equation (3), the first bending resonant frequency of the gas is 4774.1 Hz.

### 2.2. Finite-Element Method (FEM)

**Figure 3:**
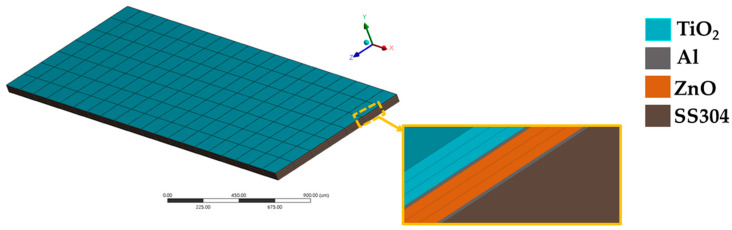
Mesh used in the FEM model of the gas sensor.

Figure 3 shows the mesh of the FEM model of the gas sensor developed using ANSYS Student 2024 R2. Table 1 shows the material properties used in this FEM model.

The aluminum contacts, layers of 304 stainless steel, and ZnO and TiO_2_ layers were included in the FEM model. Table 4 indicates the piezoelectric matrix and the piezoelectric dielectric matrix of the ZnO thin film which was used in the FEM model.

## 3. Results and Discussion

**Figure 4:**
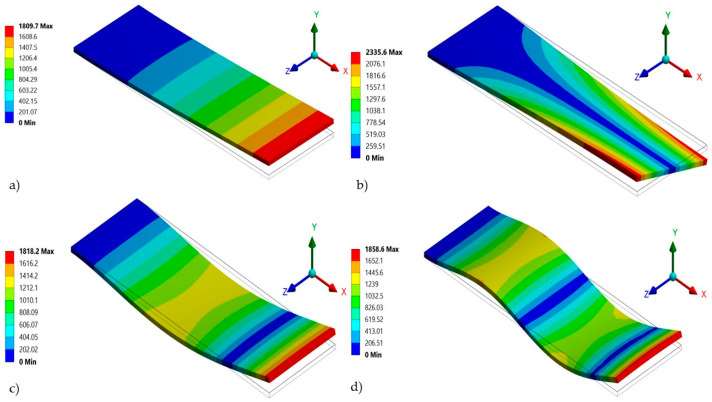
(**a**) First (4722.4 Hz), (**b**) second (28,858 Hz), (**c**) third (29,476 Hz), and (**d**) fourth (82,591 Hz) of the MEMS-based acetaldehyde gas sensor.

For the modal analysis, the FEM model of the sensor structure included a multilayer beam with a clamped end. Using this FEM model, the four vibration modes of the sensor structure were obtained (Figure 4). The first out-of-plane bending mode of the sensor has a resonant frequency of 4722.4 Hz (Figure 4a). Figure 4b,c show that the second and third vibration modes of the sensor resonator have frequencies of 28,858 Hz and 29,476 Hz, respectively. Finally, the fourth vibration mode (Figure 4d) of the sensor resonator has a frequency of 82,591 Hz.

**Figure 5:**
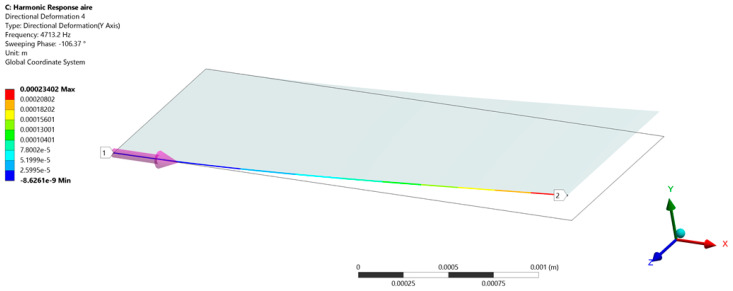
Harmonic response analysis (displacement) of the MEMS-based acetaldehyde gas sensor.

The first resonant frequency of the sensor obtained using the analytical model (Equation (3)) has a relative error of 1.08% to that of the FEM model. This relative error is small because our analytical model considered the effect of the different layers of the sensor resonator. The gas sensor has a sensitivity of 8.22259 kHz/g and the ability to detect a minimum mass variation of 2432.32 ng, which corresponds to a minimum detectable concentration of acetaldehyde of 102 ppm. These values were calculated using the previous Equations (17) and (18). To include the acetaldehyde concentration in the FEM model, a film of acetaldehyde particles was considered on all of the upper surface of the resonator. This film represents the mass of the acetaldehyde particles. A harmonic response analysis of the sensor is implemented considering the air damping and gas mass distributed on the surface of the resonator. For this analysis, the quality factor and damping ratio were 3403.30 and 14.692 × 10^−5^, respectively (Figure 5).

**Figure 6:**
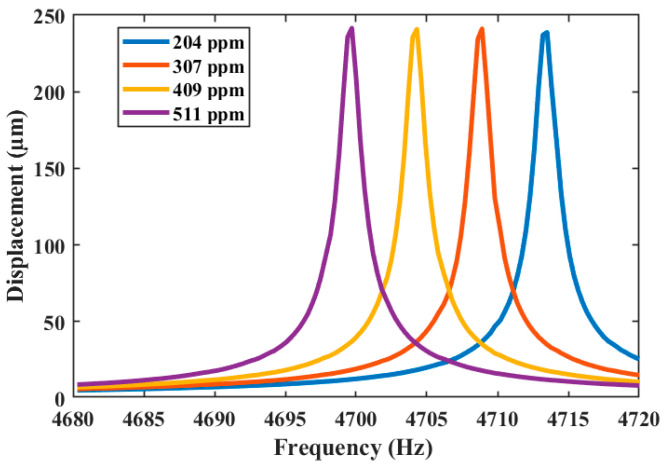
Displacement as a function of frequency of the MEMS-based acetaldehyde gas sensor.

**Figure 7:**
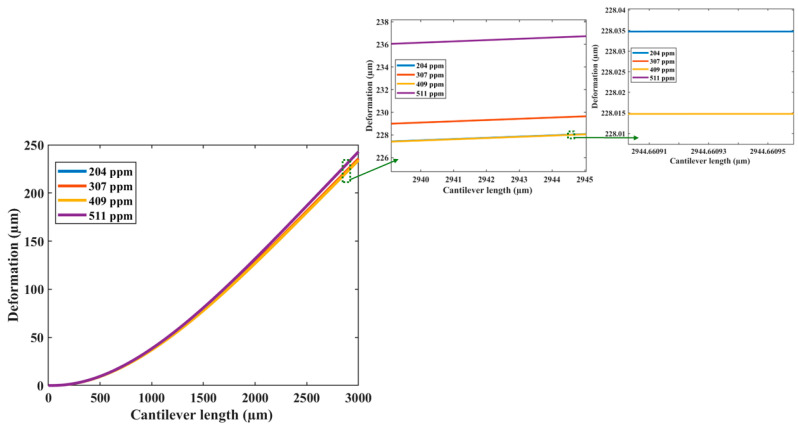
Variation of the deformation of the resonator of the MEMS-based acetaldehyde gas sensor along its length due to four different acetaldehyde concentrations.

**Figure 8:**
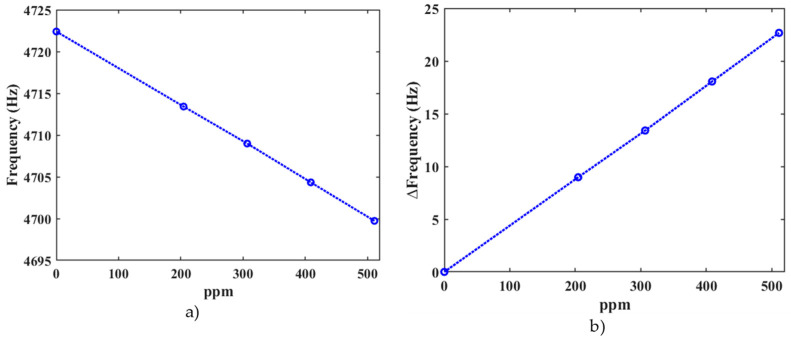
(**a**) First bending resonant frequency and (**b**) variation of first bending resonant frequency of the MEMS-based acetaldehyde gas sensor as a function of the acetaldehyde concentration.

In the harmonic response analysis, the results of the displacement of the resonator as a function of its frequency were obtained (Figure 6). Four different acetaldehyde concentrations (204 ppm, 306 ppm, 408 ppm, and 511 ppm) were considered. The first bending resonant frequency of the sensor decreases when the acetaldehyde concentration increases. Figure 7 shows the absolute values of the deformation of the resonator along its length considering the four different acetaldehyde concentrations. Figure 8 depicts the first bending resonant frequency of the gas sensor as a function of the acetaldehyde concentration. To calculate the sensor sensitivity, we used Equations (19) and (20). The sensor has a first bending resonant frequency of 4722.4 Hz without considering the acetaldehyde concentration in the FEM model. When the acetaldehyde concentration is included in the FEM model, the first bending resonant frequency of the sensor decreases. Thus, the variation in the first bending resonant frequency of the gas sensor increases when the acetaldehyde concentration increases. 

Table 5 depicts the main results of the MEMS-based resonant and acetaldehyde gas sensor. 

## 4. Conclusions

The design of an acetaldehyde gas sensor based on MEMS technology was reported. The sensor design considered an AISI 304 steel beam, a zinc oxide film, and a titanium oxide film. The analytical modeling of the mechanical behavior of the gas sensor was proposed using the methods of Rayleigh and Macaulay, as well as the Euler–Bernoulli beam theory. This analytical modeling incorporated the effect of all the layers of the gas resonator on its first bending resonant frequency and deflection. In addition, FEM models of the gas sensor were developed to predict its mechanical behavior under different acetaldehyde concentrations. The first bending resonant frequency of the gas sensor obtained using the FEM model agreed well with respect to the analytical model. The gas sensor had a first bending resonant frequency of 4722.4 Hz, a minimum sensitivity of 8.22259 kHz/g, and a minimum detectable concentration of acetaldehyde of 2432.32 ng (102 ppm). The gas sensor could be used in homes for monitoring acetaldehyde concentrations in real time and at room temperature. Thus, this sensor could be integrated with an alarm system to detect high acetaldehyde concentrations in the surroundings.

## Figures and Tables

**Table 1:** Properties of the materials used in the acetaldehyde gas sensor [25,26,27,28,29].

Geometric Parameter	Material	Young’s Modulus E (GPa)	Poisson Ratio ν	Density ρ (kg/m^3^)
*b*_11_	304 stainless steel (SS304)	2 × 10^11^	0.29	8000
*b*_21_, *b*_41_	Aluminum	7 × 10^10^	0.331	2700
*b*_31_	ZnO	1.37 × 10^11^	0.25	5665
b_51_	TiO_2_	1.51 × 10^11^	0.27	3840

**Table 2:** Geometric parameters of the gas sensor.

Geometric Parameter	Dimension (mm)
*L_S_*_1_	3
*b*_11_ = *b*_21_ = *b*_31_ = *b*_41_	1
*h*_11_	0.05
*h*_21_	1 × 10^−4^
*h*_31_	1 × 10^−3^
*h*_41_	1 × 10^−3^

**Table 3:** Bending stiffness, reaction load (*R*_0_), and bending moment (*M*_0_) of the equivalent gas sensor.

Parameter	Magnitude
*EI_z_*	2.441 × 10^−6^ N·m^2^
*ω_si_*	3.958 × 10^−3^ N·m^−1^
*M*_0_	1.781 × 10^−8^ N·m
*R*_0_	2.1 × 10^−5^ N

**Table 4:** Material properties of the piezoelectric ZnO thin film used in the FEM model of the acetaldehyde gas sensor [26].

ZnO piezoelectric stress matrix [*e*]
$\left[e\right]={\left[\begin{array}{ccc} 0 & 0 & -0.570878\\ 0 & 0 & -0.570878\\ 0 & 0 & 0.428446\\ 0 & 0 & 0\\ 0 & -0.480816 & 0\\ -0.480816 & 0 & 0 \end{array}\right]}_{\;6\times 3}\frac{C}{m^{2}}$ ZnO piezoelectric dielectric matrix [*εr*] under the constant strain $\left[\varepsilon_{r}\right]={\left[\begin{array}{ccc} 7.57 & 0 & 0\\ 0 & 7.57 & 0\\ 0 & 0 & 8.31 \end{array}\right]}_{\;3\times 3}$

**Table 5:** Comparison of operating parameters of various gas sensors.

Dimensions (µm)	Freq. (Hz)	Q Factor	Sensibility (ppm)	Sensitive Material	Sensing Temperature °C	Ref.
N.A.	N.A.	N.A.	20 to 100	p-n heterojunction interface of NiO nanosheets into WO_3_ nanorods	250	[15]
N.A.	N.A.	N.A.	5 to 50	Co-doped (1−5 wt%) ZnO films	26.85	[16]
N.A.	N.A.	N.A.	1 to 100	Polystyrenesulfonate (PEDOT:PSS) doped with MoS_2_ quantum dots (QDs).	Room temperature	[17]
35 × 25 × 5	11,100,000	4730	4.8 to 3600	Polyglycolic acid	Room temperature	[18]
300 × 1000	74,800	-- *	25.1	AlN film	24	[19]
60 × 30 sense-plate supported by two 125 × 5 microbeams	36,000	-- *	1	P25DMA and PANI.	Room temperature	[20]
3000 × 1000 × 52.2	4722.4	3403.305	102	Film TiO_2_	Room temperature	This work

* Data not available in the literature. N.A. Not applicable.

## Data Availability

Most of the steps and details have been provided in the manuscript. However, more detail and information can be obtained from the authors.
